# Alleged Discharge of Hairs from the Hand

**Published:** 1842-01

**Authors:** 


### THE ALLEGED DISCHARGE OF HAIRS FROM THE HAND.

The Lexington papers announce that Miss Penelope Stout's case is a hoax. The young Miss was detected sticking the hairs and bristles under the thumb nail.

